# Synthesis and Characterisation of Photocrosslinked poly(ethylene glycol) diacrylate Implants for Sustained Ocular Drug Delivery

**DOI:** 10.1007/s11095-017-2298-9

**Published:** 2018-01-16

**Authors:** Kathryn McAvoy, David Jones, Raghu Raj Singh Thakur

**Affiliations:** 1 School of Pharmacy, Queen’s University Belfast, 97 Lisburn Road, Belfast, Northern Ireland BT9 7BL UK; 2 School of Pharmacy, Medical Biology Centre, Queen’s University Belfast, 97 Lisburn Road, Belfast, BT9 7BL UK

**Keywords:** ocular drug delivery, photocrosslinked, poly(ethylene glycol) diacrylate, protein delivery, triamcinolone acetonide

## Abstract

**Purpose:**

To investigate the sustained ocular delivery of small and large drug molecules from photocrosslinked poly(ethylene glycol) diacrylate (PEGDA) implants with varying pore forming agents.

**Methods:**

Triamcinolone acetonide and ovalbumin loaded photocrosslinked PEGDA implants, with or without pore-forming agents, were fabricated and characterised for chemical, mechanical, swelling, network parameters, as well as drug release and biocompatibility. HPLC-based analytical methods were employed for analysis of two molecules; ELISA was used to demonstrate bioactivity of ovalbumin.

**Results:**

Regardless of PEGDA molecular weight or pore former composition all implants loaded with triamcinolone acetonide released significantly faster than those loaded with ovalbumin. Higher molecular weight PEGDA systems (700 Da) resulted in faster drug release of triamcinolone acetonide than their 250 Da counterpart. All ovalbumin released over the 56-day time period was found to be bioactive. Increasing PEGDA molecular weight resulted in increased system swelling, decreased crosslink density (Ve), increased polymer-water interaction parameter (χ), increased average molecular weight between crosslinks (Mc) and increased mesh size (ε). SEM studies showed the porosity of implants increased with increasing PEGDA molecular weight. Biocompatibility showed both PEGDA molecular weight implants were non-toxic when exposed to retinal epithelial cells over a 7-day period.

**Conclusion:**

Photocrosslinked PEGDA implant based systems are capable of controlled drug release of both small and large drug molecules through adaptations in the polymer system network. We are currently continuing evaluation of these systems as potential sustained drug delivery devices.

## Introduction

Delivery of drug molecules to treat visually impairing ocular diseases, namely those that originate in the posterior segment of the eye, has been an extremely challenging task for pharmaceutical scientists and retinal specialists. The most prevalent posterior segment ocular conditions that affect vision are age-related macular degeneration (AMD) and diabetic retinopathy (DR). AMD results in damage to the macula, the central area of the retina, a zone that is essential for detailed and central vision. The condition is extremely detrimental to those affected as it results in the inability to carry out normal, everyday activities due to the disruption of central vision. AMD is the leading cause of irreversible blindness in the Western world (1–4), estimates show 8.7% of the global population are suffers (5) and the World Health Organisation state that 8 million people have severe blindness due to AMD (6). The development of DR is common in poorly controlled diabetic patients. Over time consistently raised blood glucose levels can cause narrowing and leaking of blood vessels within the posterior segment of the eye. This results in retinal damage and ultimately blindness if the condition is left untreated. There is estimated to be 93 million patients suffering with DR worldwide (7).

The treatment of both AMD and DR involves the delivery of large molecular weight (MW) protein based therapeutics (e.g. anti-vascular endothelial growth factors (VEGF)), which aims to prevent the choroidal neovascularisation (CNV) associated with these conditions. In addition, small MW corticosteroid molecules are also used to reduce the inflammation associated with AMD and DR, and theoretically they can also benefit patients by acting as anti-angiogenic and anti-fibrotic agents and stabilising the blood retinal barrier (8). Delivery of these agents to the required site of action is problematic, as the eye contains multiple barriers designed for protection and to aid in preventing unwanted substances entering the ocular tissue. This results in many challenges associated with the delivery of both small and larger MW drug molecules into the eye and profusion to the intended area of action.

The posterior segment cannot be treated effectively using topical formulations, as they are incapable of reaching the required site of action. Consequently intravitreal injections have become the standard delivery method, but due to their invasive nature multiple adverse effects are associated (9). The limitations of current delivery methods have led to increased research into controlled release systems for ocular delivery. A significant emerging field of research is implantable polymer based systems, which aim to provide sustained release of drug molecules in the back of the eye. There are three polymer based ocular implants currently on the market; Retisert® and Iluvein® are non-biodegradable systems that provided sustained delivery of fluocinolone acetonide for up to 3 years (10). The only biodegradable system available is Ozurdex® that releases dexamethasone over a 6 month time period (10). At present no ocular controlled release system has demonstrated the ability to adequately deliver peptide or protein molecules to the posterior segment. This is a major problem as multiple therapies indicated to treat posterior segment disorders involve the use of protein-based molecules.

Photocrosslinked implant systems have been exploited for multiple applications over the past decades. Their use in biomedical applications such as tissue engineering (11,12) and prevention of thrombosis (13,14) is well documented. In terms of use in drug delivery (15,16) these photocrosslinked systems have shown the ability to provide drug release for markedly extended time periods.

Poly(ethylene glycol) (PEG) based polymers are used in a variety of biomedical applications as they can be easily synthesised and exhibit good biocompatibility and tissue-like properties (17). Specifically, PEG based polymers containing acrylate side groups show great potential in the area of photocrosslinking. PEG diacrylate (PEGDA) contains double-bond acrylate groups at each end of the PEG chain, giving it the ability to undergo free radical photo-polymerisation in the presence of a photoinitiator molecule (18) to produce a photocrosslinked system or implant (11). PEGDA is non-toxic, elicits only a minimal immune response (19,20) and is a biodegradable polymer. Browning *et al*. recently demonstrated that the primary mechanism of PEGDA hydrogel degradation *in vivo* is due to hydrolysis of the end group acrylate esters linkages within the polymer (21). PEGDA is available in various MWs making it ideal for obtaining the required system crosslink density (18) and its highly versatile nature allows for small adaptions to optimise the system (21).

As PEGDA has been proven to be a favorable material for biological applications (25) and is used in the engineering of a number of tissues such as bone (26), cartilage (27) and cornea (28,29), in this study we investigate photocrosslinked PEGDA hydrogels for their use in drug delivery systems. The tunable nature of photocrosslinked PEGDA hydrogels systems and their accepted use in biomedical applications makes them ideal candidates for adaption to provide a controlled drug release. Specifically, photocrosslinked systems, such as those composed of PEGDA, have advantages in the release of protein drug molecules. Most significantly initiation of photocrosslinkage does not require extreme pH conditions, organic solvents or high temperature (30), which could cause issues when working with protein molecules.

This study aims to fabricate and characterise PEGDA-based photocrosslinked implants of different polymer MW (250 and 700 Da) for sustained ocular delivery of triamcinolone acetonide (TA, 434 Da) and ovalbumin (OVA, 45 kDa). Alternations in the MW of PEGDA results in changes to the crosslink density of the system formed, with lower MWs resulting in a more densely crosslinked system. This study has selected PEGDA at MW 250 and 700 Da for investigate to assess the degree of difference in release profile and implant properties at these MWs. With regards to drug molecule selection, TA has been chosen for its clinical relevance in treating AMD and DR, while OVA was chosen as a model protein molecule that is similar in MW to anti-VEGF drug ranibizumab (Lucentis®). *In vitro* release profiles from the PEGDA implants were investigated. Due to the vast difference in MW of each drug, a number of pore forming agents have been incorporated into the PEGDA implants to assess their effect on pore formation, drug release, protein bio-stability, mechanical properties, swelling behaviour, network parameters and biocompatibility.

### Materials

PEGDA with MW of 250 Da and 700 Da (hereafter referred as PEGDA 250 and PEGDA 700), 1-[4-(2-hydroxyethoxy)-phenyl]-2-hydroxy-2-methyl-1-propanone (Irgacure® 2959), OVA, mannitol, sodium bicarbonate, sodium carbonate, gelatin, maltose, sucrose, sodium chloride, monoclonal anti-chicken egg albumin produced in mouse, tetramethylbenzidine liquid (TMB) substrate, hydrochloric acid and HPLC grade acetonitrile were purchased from Sigma-Aldrich (Basingstoke, England). TA was purchased from Spruyt Hillen bv (Ijsselstein, The Netherlands). Scotch™ Magic tape was purchased from 3 M, Berkshire UK. Phosphate buffered saline (PBS), HPLC grade water and all other chemicals used were of analytical grade. 27G needles were purchased from Terumo UK Ltd. (Surrey, United Kingdom). 1 mL disposable medical syringes purchased from Becton, Dickinson and Company (Oxford, UK). Rabbit anti-OVA-biotin conjugate (polyclonal) was purchased from Novus Biologicals (Cambridge, United Kingdom). Streptavidin-Horse radish peroxidase conjugate was purchased from BioLegend® (San Diego, United States). Superblock T20 buffer was purchased from Thermo Scientific Pierce (Rockford, United States). DAPI (4′,6-Diamidino-2-Phenylindole, Dihydrochloride) solution (Fisher Scientific, Loughborough, UK).

## Methods

### Fabrication of PEGDA Implants

The drugs under investigation (TA and OVA) were incorporated in PEGDA (700 or 250) to produce 2.5% *w*/w solutions. Once the drug was fully dissolved/suspended the desired amount of pore former was then added to the drug/PEGDA (700 or 250) solutions and left to dissolve at room temperature. Prior to photocrosslinking, Irgacure® 2959 (2% *w*/*v* in 70% ethanol in water) was added to the formulation and vortexed for 1 min to ensure complete mixing. The composition of all prepared formulations are shown below in Table I.

**Table I:** Composition of PEGDA 700 and PEGDA 250 Photocrosslinked Implants

Formulation code	Drug loaded at 2.5% *w*/w	Pore former loaded at 10% w/w	PEGDA MW
T1	TA	–	700
T2	TA	–	250
T3	TA	Mannitol	700
T4	TA	Mannitol	250
T5	TA	NaHCO_3_	700
T6	TA	NaHCO_3_	250
T7	TA	NaCO_3_	700
T8	TA	NaCO_3_	250
T9	TA	Gelatin	700
T10	TA	Gelatin	250
T11	TA	Maltose	700
T12	TA	Maltose	250
T13	TA	Sucrose	700
T14	TA	Sucrose	250
T15	TA	NaCl	700
T16	TA	NaCl	250
O1	OVA	–	700
O2	OVA	–	250
O3	OVA	Mannitol	700
O4	OVA	Mannitol	250
O5	OVA	NaHCO_3_	700
O6	OVA	NaHCO_3_	250
O7	OVA	NaCO_3_	700
O8	OVA	NaCO_3_	250
O9	OVA	Gelatin	700
O10	OVA	Gelatin	250
O11	OVA	Maltose	700
O12	OVA	Maltose	250
O13	OVA	Sucrose	700
O14	OVA	Sucrose	250
O15	OVA	NaCl	700
O16	OVA	NaCl	250
C1	–	–	700
C2	–	–	250
C3	–	Mannitol	700
C4	–	Mannitol	250
C5	–	NaHCO_3_	700
C6	–	NaHCO_3_	250
C7	–	NaCO_3_	700
C8	–	NaCO_3_	250
C9	–	Gelatin	700
C10	–	Gelatin	250
C11	–	Maltose	700
C12	–	Maltose	250
C13	–	Sucrose	700
C14	–	Sucrose	250
C15	–	NaCl	700
C16	–	NaCl	250

**Fig. 1:**
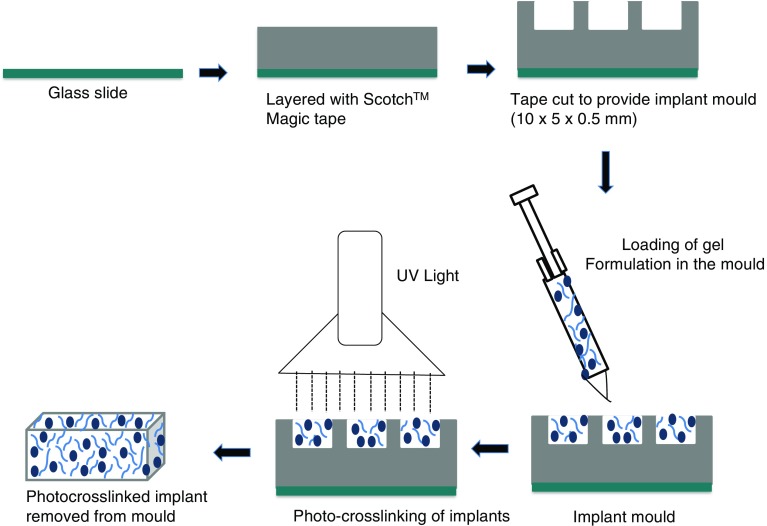
Schematic representation demonstrating PEGDA implant fabrication.

Moulds for implant fabrication were then prepared using Scotch™ Magic tape (one strip has a thickness of 50 μm) layered onto glass microscope slides and cut to the required implant dimensions of 10 × 5 × 0.5 mm (Fig. 1). The previously prepared formulations (Table I) where then loaded into the moulds using a 27G needle. Each mould was passed through the UV crosslinker, Fusion UV Light-Hammer 6 high power UV curing system (Maryland, USA) fitted with a “D” class mercury discharge bulb, with a belt speed of 10–10.5 m/s and at wavelength 365 nm, lamp intensity 50% and 5 times to crosslink the formulation. Each implant was then carefully removed from their mould and weighed.

### *In vitro* Drug Release Studies

For *in vitro* drug release studies, TA loaded implants were placed in a plastic tub containing 50 mL PBS (pH 7.4 ± 0.2) release media to maintain sink conditions. All the experiments were carried out in triplicate. The tubs were then incubated at 37°C and 40 RPM and at predetermined time intervals the entire medium was removed and replaced with fresh medium. The concentration of released TA in the PBS samples was then analysed using the HPLC assay described below.

For *in vitro* drug release study of OVA each preformed implant was placed in a glass vial containing 5 mL PBS (pH 7.4 ± 0.2) release media and samples as above. The concentration of released OVA in the PBS samples was then analysed using the size-exclusion HPLC assay described below.

### Analytical Techniques

Analysis of TA samples was carried out using reversed-phase Agilent 1260 Infinity Quaternary System HPLC using an Agilent Zorbax Eclipse Plus 250 mm, 4.6 mm ID, 5 μm particle size, C18 bonded silica column and an Agilent Zorbax guard column held at 25°C (Agilent Technologies Ltd., Stockport, UK). A mobile phase of 60% water and 40% acetonitrile at UV absorbance of 236 nm was found to give optimal peak shape. Injection volume was fixed to 20 μl and flow rate was set at 0.8 mL/min. A standard calibration curve was then prepared, between the concentration range of 0.1–250 μg/mL, and used to determine the TA drug concentration in each of the release samples.

Analysis of OVA samples was carried out using Agilent 1260 Infinity Quaternary System HPLC using a Phenomenex BIOSEP-SEC-s3000 300 mm, 7.8 mm ID, 3 μm particle size column and GFC 3000 guard column held at 25°C (Phenomenex., Cheshire, UK). A mobile phase of 250 mM NaCl at UV absorbance of 214 nm was found to give optimal peak shape. Injection volume was fixed to 20 μL and flow rate was set at 1 mL/min. A standard calibration curve was then prepared, between the concentration range 3.125–100 μg/ml, and used to determine the OVA drug concentration in each of the release samples.

### ELISA for Bioactivity Analysis of OVA

The bioactivity of released OVA was determined using a sandwich ELISA technique previously developed by McCrudden *et al*. (31). Monoclonal anti-chicken egg ovalbumin antibody produced in mouse (MoAb) was diluted in 0.1 M bicarbonate buffer, pH 9.6 to the optimised concentration of 2.5 μg/mL. An aliquot (100 μl) of this anti-ovalbumin was dispensed into the plate and incubated overnight at 4°C. The plate washed to remove unbound antibody (0.05% Tween 20 in PBS) 3 times. Blocking was performed with SuperBlock**®** T20 (200 μl/well), for 2 h. A 50 μl volume of sample was dispensed into each well, with each sample analysed in triplicate and a freshly prepared standard concentration curve was plated (50–1000 ng/ml), and incubated for 1 h. Excess sample was washed off (as before). Following this, 50 μl of rabbit anti-chicken egg ovalbumin, polyclonal antibody conjugate with biotin at the optimised concentration of 1 μg/mL in SuperBlock**®** T20 buffer was added to each well for 1 h. Excess sample was washed off (as previously detailed). The plate was then incubated with Streptavidin – Horseradish Peroxidase (Strep-HRP) conjugate at the optimised dilution of 1:2000 in PBS, for 30 min. Washing was performed as before. To detect the antibody binding, 100 μl of substrate TMB (3,3′,5,5′-tetramethylbenzidine) was added to each well and incubated for 20 min and the reaction was ended using 100 μl/well, 4.0 M hydrochloric acid. Optical density was measured at 450 nm using a micro 96 well plate spectrophotometer (Powerwave™ XS, Bio-Tek Instruments Inc., Minooski, USA). Results were expressed as means ± SD of three replicates.

### Dynamic and Equilibrium Swelling Studies

For swelling studies the crosslinked implants were weighed as m_o_ (xerogels) and were then swollen in HPLC grade water for 48 h at 37°C. At regular intervals, the implants were removed, blotted with filter paper to eliminate excess surface water and weighed as m_t_ (hydrogels). The implants at equilibrium were weighed as m_e_, and were dried under vacuum at 80°C for 24 h to obtain extracted xerogels, which were weighed as m_d_ (32). The percentage swelling, % S, and equilibrium water content, EWC, was calculated, respectively, by using Eq. 1 and 2.

1 $$ \% Swelling=\left(\frac{Mr- Mo}{Mo}\right)\ x\ 100 $$

2 $$ \% EWC=\left(\frac{Mo- Md}{Md}\right)\ x\ 100 $$

### Network Parameters

M_c_, the average MW between crosslinks, is calculated using Flory-Rehner theory, which gives the values of Mc between two adjacent crosslinks and represents the degree of crosslinking of hydrogel networks (33) and is shown in Eq. 3. The magnitude of Mc affects the mechanical, physical and thermal properties of crosslinked polymers, as we descried previously (32).

3 $$ {M}_c=\frac{-{d}_p{V}_s{\varnothing}^{\raisebox{1ex}{$1$}\!\left/ \!\raisebox{-1ex}{$3$}\right.}}{\left[\ln \left(1-\varnothing \right)+\varnothing +\chi\ {\varnothing}^2\right]} $$

V_s_ is the molar volume of water (18 cm^3^/mol), Φ is volume fraction of polymer in the hydrogel, χ is the Flory–Huggins polymer–solvent interaction parameter.

The volume fraction of a polymer, Φ, in the swollen state defines the amount of liquid that can be enter a hydrogel and is described as a ratio of the polymer volume to the swollen gel volume (32), show in Eq. 4.

4 $$ \varnothing ={\left(1+\frac{d_p}{d_s}\ \left[\frac{m_a}{m_b}\right]-1\right)}^{-1} $$

Where, m_a_ and m_b_ are the mass of polymer before and after swelling and d_p_ and d_s_ are the densities of polymer and solvent, respectively. The density of the polymeric films was calculated using the following formula; d_p_ = w/SX, where; X is the average thickness of the film, S is the cross-sectional area and w weight of the film (34).

The polymer–water interaction parameter (χ) of hydrogels can be obtained experimentally *via* Eq. 5.

5 $$ \chi =\frac{1}{2}+\frac{\varnothing }{3} $$

Crosslink density, Ve, was determined using Eq. 6. Ve represents the number of elastically effective chains, totally induced in a perfect network, per unit volume (32). Where N_A_ is Avagadro’s number (6.023 × 10^23^ mol^−1^) (32).

6 $$ {V}_e={d}_p{N}_A/{M}_c $$

Mesh size was calculated first by assessing the distance between polymer chains, r_0_^−2^. Where l is the average bond length, 1.54, Cn is the characteristic ratio for the polymer here we will use the Cn of PEG, typically 4.0 (35,36).

7 $$ {\left({r_o}^{-2}\right)}^{\frac{1}{2}}=l{(n)}^{\frac{1}{2}}\ {C_n}^{\frac{1}{2}} $$

The mesh size, ε, was then calculated by:

8 $$ \varepsilon ={\varnothing}^{\frac{1}{3}}\ {\left({r_o}^{-2}\right)}^{-\frac{1}{2}} $$

### Mechanical Strength

**Fig. 2:**
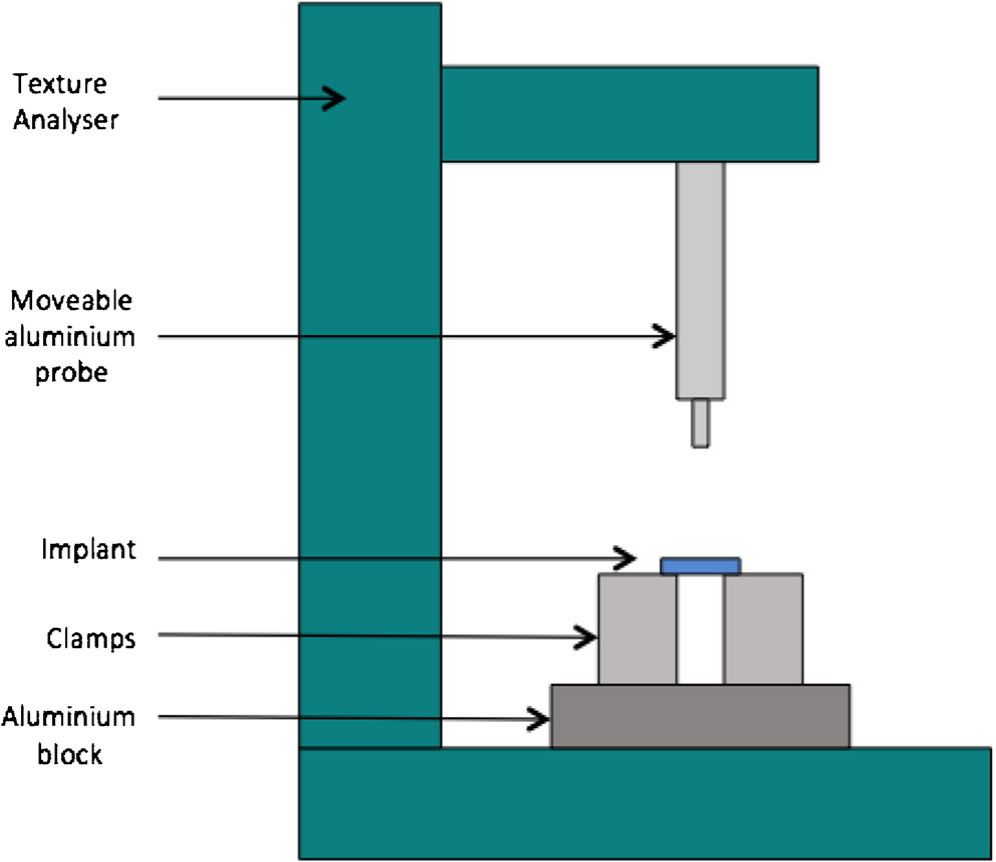
Illustration of texture analyser set up from implant compression.

Addition of pore forming agents on the implant mechanical property was assessed *via* the comparison of implant strength and integrity. This was conducted using a TA.XTplus Texture Analyser (Stable Micro Systems, Surrey, UK) to compress the implants 5 mm, as shown in Fig. 2. The implants were tested directly after fabrication (dry implants) and then again after 24 h in PBS (pH 7.3 ± 0.2) (wet implants). Implants were attached to the probe and the Texture Analyser was set in compression mode, and the probe moved towards a solid aluminium block. When the implant came in contact with the solid surface, the test began with the probe moving at 0.05 mm/s. The maximum force required to compress the implant to 5 mm was recorded using the Exponent TA.XT software (Version 4). The study was performed in triplicate for each formulation.

### Structural Analysis using Scanning Electron Microscope (SEM)

In order to investigate the structural differences in implants composed of varying PEGDA molecular weights and assess the addition of pore forming agents, SEM images were collected using a Hitachi TM 3030 Table top Microscope (Hitachi High-Technologies Europe GmbH, Krefeld Germany). After photocrosslinking and placement in PBS (pH 7.3 ± 0.2) for both 24 h and 7 days the implants were then removed and rinsed using deionised water, with the excess liquid removed using tissue paper, the implants were left to dry. Once dry, the implants were sectioned accordingly and mounted for imaging.

### Biocompatibility Testing

Biocompatibility experiments were carried out by both indirect and direct contact methods. During formulation preparation both PEGDA 700 and PEGDA 250 were filtered through a sterile 0.2 μm syringe filter with a membrane material of cellulose acetate (VWR®, International Ltd., Leicestershire, UK). Prepared formulations where then loaded into the moulds and crosslinked using a UV crosslinker, Fusion UV LightHammer 6 high power UV curing system (Maryland, USA) fitted with a “D” class mercury discharge bulb, with a belt speed of 9.8-10 m/s and at wavelength 365 nm, lamp intensity 50% and 5 times to crosslink the formulation.

#### Indirect Contact Assay

Each implant was then carefully removed from their mould, dipped in ethanol to sterilise and placed into 5 mL DMEM/F-12 media (Gibco®, Life Technologies™, Paisley UK) in autoclaved glass vials. At defined time points (1, 4 and 7 days after formation), the entire 5 mL of media was removed and replaced with fresh media. The media collected at various time points was then exposed to the cells. All treatments were performed on human retinal pigment epithelia cells (ARPE-19) seeded in 96-well plates (Nunc®, Denmark) at a cellular density of 2 × 10^4^ cells/well, which were incubated at 37°C for 24 h in DMEM/F-12. The DMEM/F-12 medium was removed and replaced with 200 μl of the release media from each time point (fresh media was used as the control). Subsequently, the cells were incubated for a further 24 h.

#### Direct Contact Assay

Each implant was removed from their mould, cut to size, dipped in ethanol to sterilise and placed into 5 mL DMEM/F-12 media (Gibco®, Life Technologies™, Paisley UK) in autoclaved glass vials and incubated at 37°C until swollen to equilibrium. Simultaneously, ARPE-19 cells were seeded in 96-well plates (Nunc®, Denmark) at a cellular density of 2 × 10^4^ cells/well and incubated at 37°C for 24 h in DMEM/F-12. Media was then removed from each well and implants were carefully placed on top of the cells, followed by addition of 200 μl of fresh media. The cells were then incubated for a further 24 h. Following aspiration of the remaining media, the 96-well plate was inverted to allow removal of each implant from the treatment wells.

For both indirect and direct contact methods cell viability was then determined using a MTS assay and DAPI (4′,6-diamidino-2-phenylindole) fluorescent staining.

For the cell proliferation assay 20 μl of Promega G3580 MTS assay solution (Promega Corporation, Wisconsin, USA) was added to each well. After 2 h of incubation, the UV absorbance was determined at 490 nm. The percentage cell viability was then calculated by dividing the average absorbance value of each formulation by the average absorbance of the control (which consisted of ARPE-19 cells grown in media, without any exposure to PEGDA implants) and multiplying by 100.

For DAPI fluorescent staining 50 μl of Formalin was used to fix the cells before the addition of 50 μl DAPI solution (Fisher Scientific, Loughborough, UK) diluted 2:10,000 in PBS, which was subsequently aspirated from the cells after 10 min and replaced with 50 μl of PBS, the cells were then imaged using a fluorescent microscope under the DAPI filter.

### Statistical Analysis

The effects of PEGDA MW and the loading of various pore forming agents on swelling, mechanical strength, cell viability and drug release from the implant formulations were analysed using a one-way analysis of variance (ANOVA) where *p* < 0.05 was taken to represent a statistically significant difference. When there was a statistically significant difference, post-hoc Tukey’s HSD tests were used. Statistical analysis was conducted using GraphPad Prism Version 6 (GraphPad Software Inc., San Diego, USA).

## Results

### *In vitro* Drug Release

**Fig. 3:**
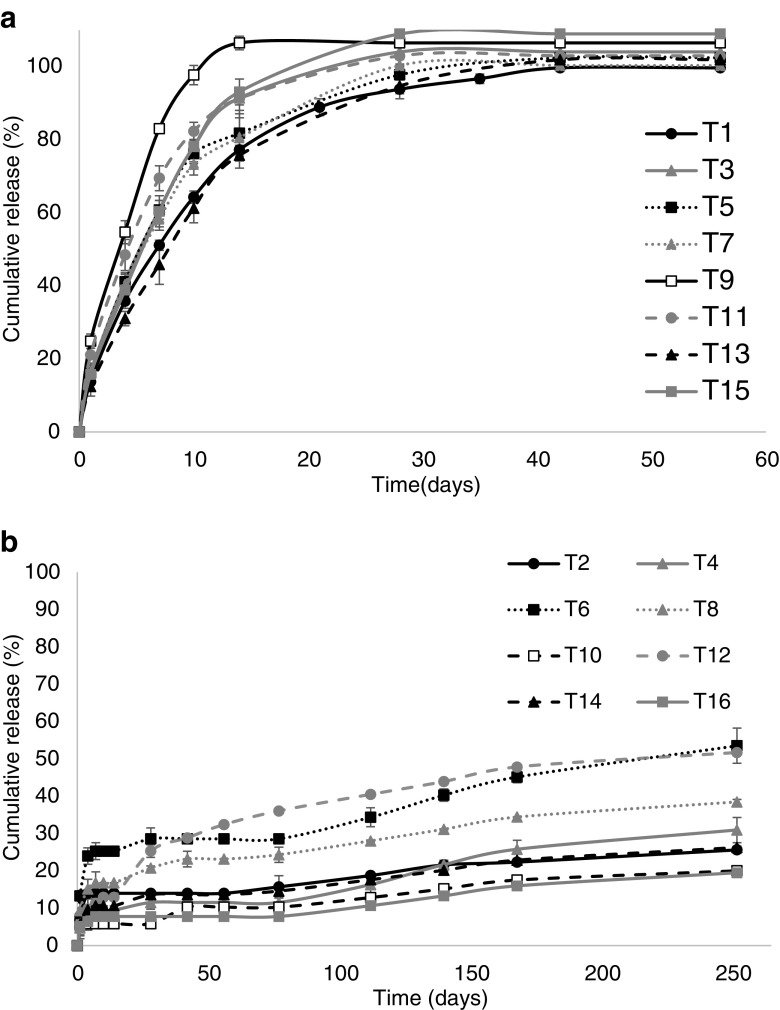
Percentage cumulative release of TA from (**a**) PEGDA 700 and (**b**) PEGDA 250 implants. (Mean ± S.D., *n* = 3).

TA release, from all PEGDA 700 implant formulations, reached approximately 100% between days 21–28. From the range of pore forming agents selected only sodium bicarbonate (T7) and sucrose (T13) display a slower release rate than the control formulation (T1) without pore forming agent, and this observed difference is not significant (*p* = 0.9971 and 0.8759 respectively). All other formulation types released TA faster than the control. However, there was not a significantly faster rate of release, with the exception of gelatin (T9). Gelatin provides a significant increase in the day 1 release (burst release) of TA, 24.82% release was exhibited compared to 13.70% from the control (*p* = 0.0437), but following this time point release did not remain significantly greater than the control (Fig. 3a).

With regards to TA release from PEGDA 250 implants (Fig. 3b); no pore-forming agent showed significantly greater release than the control formulation (T2) before the day 252 time point is reached. At day 252 loading with sodium bicarbonate (T6) resulted in the greatest drug release, 53.51%, followed by maltose (T12) at 51.69% and then sodium carbonate (T8) at 38.45%, compared to the control formulation (T2), which released 25.61% of the drug loading. Of these three pore forming agents, the release from sodium bicarbonate (T6) and maltose (T12) exhibited a statistically significant increase in release rate when compared to the control formulation (*p* = 0.0450 and 0.0465 respectively). Regardless of the pore-forming agent loaded, PEGDA 250 implants showed a significantly slower release rate than those prepared with PEGDA 700.

OVA release from all implants, regardless of PEGDA MW or pore forming loading, follow a similar release pattern. An initial burst release of drug is observed, within the first 24 h, followed by a reduction in release in the following days. Results highlight that the effect of PEGDA MW on release of OVA is less substantial than the effect of pore former loading. Only formulations loaded with sodium carbonate (O7 and O8), sodium bicarbonate (O5 and O6), gelatin (O9 and O10) and sucrose (O13 and O14) showed a significant difference in release across the two PEGDA MW’s. Conversely, the addition of pore forming agent has a significant effect on OVA release in almost all formulation types. Regardless of PEGDA MW the three pore forming agents that cause the most significant increase in OVA release over the 84-day period were sodium carbonate (O7 and O8, releasing 62.34% and 38.59% respectively), sodium bicarbonate (O5 and O6, releasing 35.79% and 46.42% respectively) and sucrose (O13 and O14, releasing 30.85% and 41.48% respectively).

Interestingly, both PEGDA 700 and PEGDA 250 implants loaded with mannitol (O3 and O4) and maltose (O11 and O12) release significantly less OVA than the control formulation (at day 1, *p* < 0.0001 and *p* = 0.0005 respectively, at day 84, *p* < 0.0001 and *p* = 0.0018 respectively).

With respect to PEGDA 700 implants sodium carbonate (O7) shows the most significant increase in OVA release (at day 1 39.95% released) when compared to control (at day 1 14.68% released) (*p* < 0.0001), followed by sodium bicarbonate (O5) (at day 1 26.40% released) (*p* < 0.0001) and then sucrose (O13) (at day 1 release 23.41% released) (*p* < 0.0011). The increase in OVA release with these three pore forming agents remains significant at day 84, with the control implant exhibiting 22.23% release compared to 62.34% from sodium carbonate (O7) (*p* < 0.0001), 35.79% from sodium bicarbonate (O5) (*p* < 0.0001) and 30.85% from sucrose (O13) (*p* < 0.0524).

From OVA loaded PEGDA 250 implants, with the exception of mannitol (O4) and maltose (O12), all remaining pore forming agents release significantly more than the control samples, across all times points (day 1 *p* < 0.0001 for sodium carbonate, sodium bicarbonate, getalin and sucrose. At day 84, *p* < 0.0001 for sodium carbonate, sodium bicarbonate, getalin and *p* = 0.0022 for sucrose).

### Bioactivity of OVA

From HPLC chromatograms it can be confirmed that only visible peaks present are attributed to OVA and the dimer molecule, no degradation products are visible eluting from the column, indicating that OVA remains stable through the study (Fig. 5). However, this observation requires confirmation and thus OVA bioactivity was tested to ensure the tertiary structure was maintained throughout the release study. Release samples from the implants were studied to determine whether the released OVA was still active and that the quantity of OVA detected from the SEC HPLC assay was structurally sound. Table II presents data relating to the percentage of active OVA released after 56 days. Results indicate that active OVA was released from the implants throughout the time period. From day 1–56, greater than 90% of the OVA released was determined to be active for all formulation types. The presence or absence of pore forming agents does not appear to play a significant role in OVA activity within PEGDA 700 or PEGDA 250 implant systems. (Figures 6 and 7)

**Table II:** Percentage of active OVA in Release Samples Determined by ELISA at Selected Time Points (Mean ± S.D., n = 3)

Formulation code	Percentage of active OVA from release samples		
	Day 1	Day 28	Day 56
O1	95.44 ± 3.20	99.53 ± 0.80	95.65 ± 3.18
O2	96.09 ± 5.24	99.47 ± 5.44	91.46 ± 6.72
O3	94.77 ± 4.77	99.65 ± 5.45	91.21 ± 7.57
O4	97.97 ± 6.86	99.17 ± 6.33	96.05 ± 3.78
O5	98.97 ± 6.87	94.53 ± 8.24	94.42 ± 6.97
O6	93.06 ± 3.07	94.70 ± 2.57	95.28 ± 14.28
O7	96.49 ± 0.75	94.94 ± 2.56	91.57 ± 8.33
O8	93.99 ± 1.71	95.53 ± 1.59	91.65 ± 1.65
O9	94.04 ± 4.18	100.01 ± 5.20	90.09 ± 4.44
O10	94.61 ± 4.18	98.86 ± 1.95	96.56 ± 0.61
O11	95.26 ± 0.92	96.61 ± 3.08	90.44 ± 2.87
O12	94.72 ± 2.98	94.09 ± 1.54	92.76 ± 6.15
O13	95.06 ± 4.64	96.86 ± 1.45	98.38 ± 2.29
O14	98.66 ± 5.71	97.39 ± 2.31	92.06 ± 3.78
O15	99.97 ± 3.18	98.11 ± 4.27	99.98 ± 5.58
O16	99.64 ± 5.87	94.57 ± 4.66	90.05 ± 3.34

### Swelling Studies

In relation to implant swelling, varying the PEGDA MW shows significant differences within the implant. PEGDA 700 implants exhibit 35–50% swelling across all formulations; in contrast no PEGDA 250 implant swells above 5%. In fact C6 (containing sodium bicarbonate) exhibited an initial drop in implant weight, which may be due to loss of uncrosslinked surface monomer, but this weight drop is not significant (*p* = 0.9537).

When directly comparing formulations containing the same pore forming agents but different MW’s of PEGDA, the difference in system swelling was highly significant. With the head to head comparison of each pore forming agent with their differing MW counterpart *p* < 0.0001 for all pore forming agents and control formulations.

With regards to swelling of PEGDA 700 implants the only pore forming agents that made a significant difference to swelling is gelatin (C9) (*p* = 0.0254). No pore forming agent loaded in PEGDA 250 implants shows a significant difference in swelling when compared to the control formulation.

### Network Parameters

As shown in Table III, pore former loading does not play a significant role in the systems network parameters. However, the crosslink density, Ve, increases notably with a decrease in PEGDA MW within the implants. The network parameters also assess the polymer-water interaction within the system. In polymer–water systems, the higher the value of χ, the weaker is the interaction between polymer and water (32). This study shows that there is a slight increase in the polymer–water interaction parameter (χ) with increased crosslink density and decrease in PEGDA MW.

**Table III:** Network Parameters of PEGDA 700 and 250 Formulations Loaded with 10% w/w of Varying Pore Forming Agents

Formulation code	Network Parameters			
	Ve X 10^22^	χ	Mc	ε
C1	4.58	0.82	1.72	0.22
C2	19.91	0.83	0.40	0.17
C3	4.58	0.82	1.79	0.23
C4	17.44	0.83	0.49	0.19
C5	4.43	0.82	1.89	0.23
C6	19.56	0.83	0.47	0.19
C7	3.98	0.81	2.45	0.26
C8	14.60	0.83	0.63	0.19
C9	4.22	0.81	1.93	0.23
C10	16.54	0.83	0.63	0.18
C11	4.27	0.82	2.05	0.24
C12	12.25	0.83	0.59	0.21
C13	4.99	0.82	1.36	0.19
C14	20.42	0.83	0.42	0.18
C15	4.57	0.82	1.85	0.23
C16	12.26	0.83	0.88	0.17

With regards to Mc and mesh size, both values increase as the MW of PEGDA within the system increases. PEGDA 700 systems have a higher average MW between crosslinks and a larger mesh size.

### Mechanical Strength

Implant mechanical strength was assessed on both dry and wetted implants (which were exposed to an aqueous environment for 24 h). All dry implant formulation types loaded with pore forming agents exhibited an increased mechanical strength when compared to the control implants (C1 and C2), containing no pore former loading. The increase in mechanical strength observed in the PEGDA 700 implants, was not statistically significant (*p* = 0.8746). Conversely, all PEGDA 250 implants loaded with pore forming agents were significantly stronger than the control (C2) formulation with the exception of sodium bicarbonate (C8) When comparing C2 and C8 *p* = 0.8964.

### SEM Images

SEM images are useful in assessing both the top and cross-sectional view of implants in intricate detail. Figure 8 highlights the smooth external and internal surfaces of control implants, containing no pore-forming agent (C1 and C2) compared to implants loaded with sodium carbonate (formulations C7 and C8). The PEGDA 700 implants are noticeably more porous than the lower MW PEGDA implants in the top sectional image. However, upon analysis of the cross-sectional images both PEGDA MW implants show significant pore formation.

With regards to drug loading on implant pore formation, Fig. 9 highlights the difference between non-drug loaded; TA loaded and OVA loaded PEGDA 700 and PEGDA 250 implants. As demonstrated in Fig. 8, the PEGDA 700 implants are noticeably more porous than their PEGDA 250 counterpart. Additionally, a slight affect on drug loaded can be observed, with non-drug loaded implants (C3 and D3) appearing more porous than those loaded with TA and OVA.

### Biocompatibility

Cell toxicity studies were carried out using ARPE-19 cells to assess potential cytotoxicity. Experiments were carried out using both indirect and direct contact assays. Indirect contact tests involved calculating the percentage cell viability after ARPE-19 cells were exposed to release media collected 1, 4 and 7 days after the implant was added to the media. Direct contact tests involved direct placement of the implant on top of the cell line and assessing the cell viability.

Percentage cell viability was calculated using an MTS assay and reading the UV absorbance. In all cases percentage cell viability was recorded as greater than 80%. Indirect exposure to these release samples for all PEGDA 700 time points and for PEGDA 250 at day 4 and 7 actually resulted in cell proliferation as indicated by a percentage cell viability of greater than 100% (Fig. 10). Direct contact resulted in greater than 90% cell viability in all cases (Fig. 11).

Cells were also stained with DAPI, which allows the live cells to be visualised under a fluorescence filter, with live cells appearing blue. Figure 12 shows the results from staining post (A) indirect contact assay and (B) direct contact assay, both images show a high portion of live cells present with morphology of the cells maintained, corroborating the cell viability results.

## Discussion

Prevalence of debilitation, sight treating ocular diseases is increasing and although currently there are drug molecules available to treat these conditions, delivery of these molecules to the required site of action is suboptimal and associated with a high level of adverse effects. Therefore, a delivery device capable of providing an extended time period for drug release could present a viable alternative to the currently used intravitreal injections. This study focuses on the development of novel photocrosslinked biodegradable ocular implants that will provide continuous sustained drug delivery to the posterior segment of the eye. Photocrosslinked implants composed of varying PEGDA MWs (250 Da and 700 Da), photoinitiator and loaded with both small and large MW drug molecules and various pore forming agents have been formulated and characterised.

**Fig. 4:**
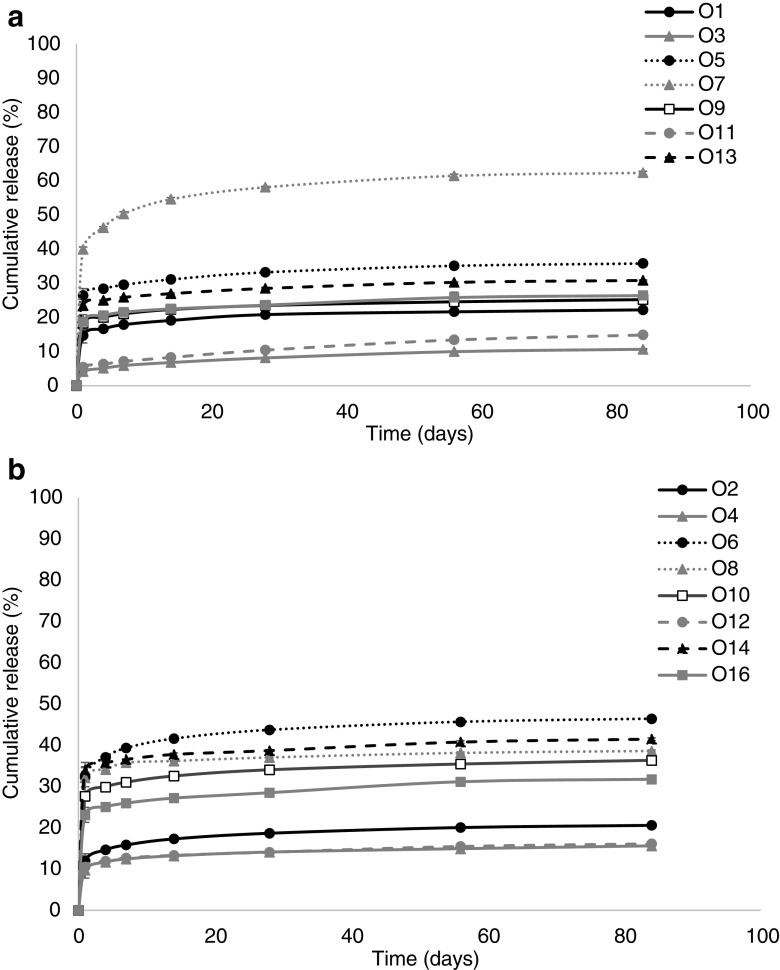
Percentage cumulative release of OVA from (**a**) PEGDA 700 and (**b**) PEGDA 250 implants. (Mean ± S.D., n = 3).

As expected, drug release results differed based on implant formulation type and was dependent on the type of drug, PEGDA MW and the pore forming agent. It was postulated that the addition of water-soluble agents would result in the formation of pores by dissolving when infiltrated by the aqueous release medium, thus causing the porogen to leach out of the implant system, in turn leaving behind a porous structure (37). This hypothesis reflected the results observed regarding OVA from PEGDA implants, with the use of specific pore forming agents showing marked increase in OVA release compared to the control formulation (Fig. 4). Namely, sodium carbonate (O7 and O8), sodium bicarbonate (O5 and O6) and sucrose (O13 and O14), were shown to be the most effective pore forming agents with respect to increasing OVA release. As stated in the results section, there were only two pore forming agents that did not improve OVA release, namely mannitol (O3 and O4) and maltose (O11 and O12), which both release significantly less OVA than the control formulation. These results indicate that there is a potential interaction between these pore forming agents and the protein molecule, which is preventing drug release (38). The effect of pore forming agent on TA release from PEGDA 700 implants appears minimal and this is likely related to the MW of the drug molecule. Due to TAs low MW the introduction of pores within the system does not produce any significant effect on release from the implant, as it already exhibits a significant level of release devoid of pore forming agents. Regarding TA loaded PEGDA 250 implants, the effect of pore former loading on drug release does become evident, but not until long-term release periods are reached.

With respect polymer MW on release rate, there is significantly higher release of TA from PEGDA 700 implants when compared to PEGDA 250 implants (*p* < 0.0001 for all pore forming agents). This is likely due the lower PEGDA MW resulting in a denser and more tightly crosslinked system inhibiting drug release and leaving the TA molecule trapped within the implant system. In contrast, the effect of PEGDA MW on drug release is less significant than the use of certain pore forming agents on OVA release.

**Fig. 5:**
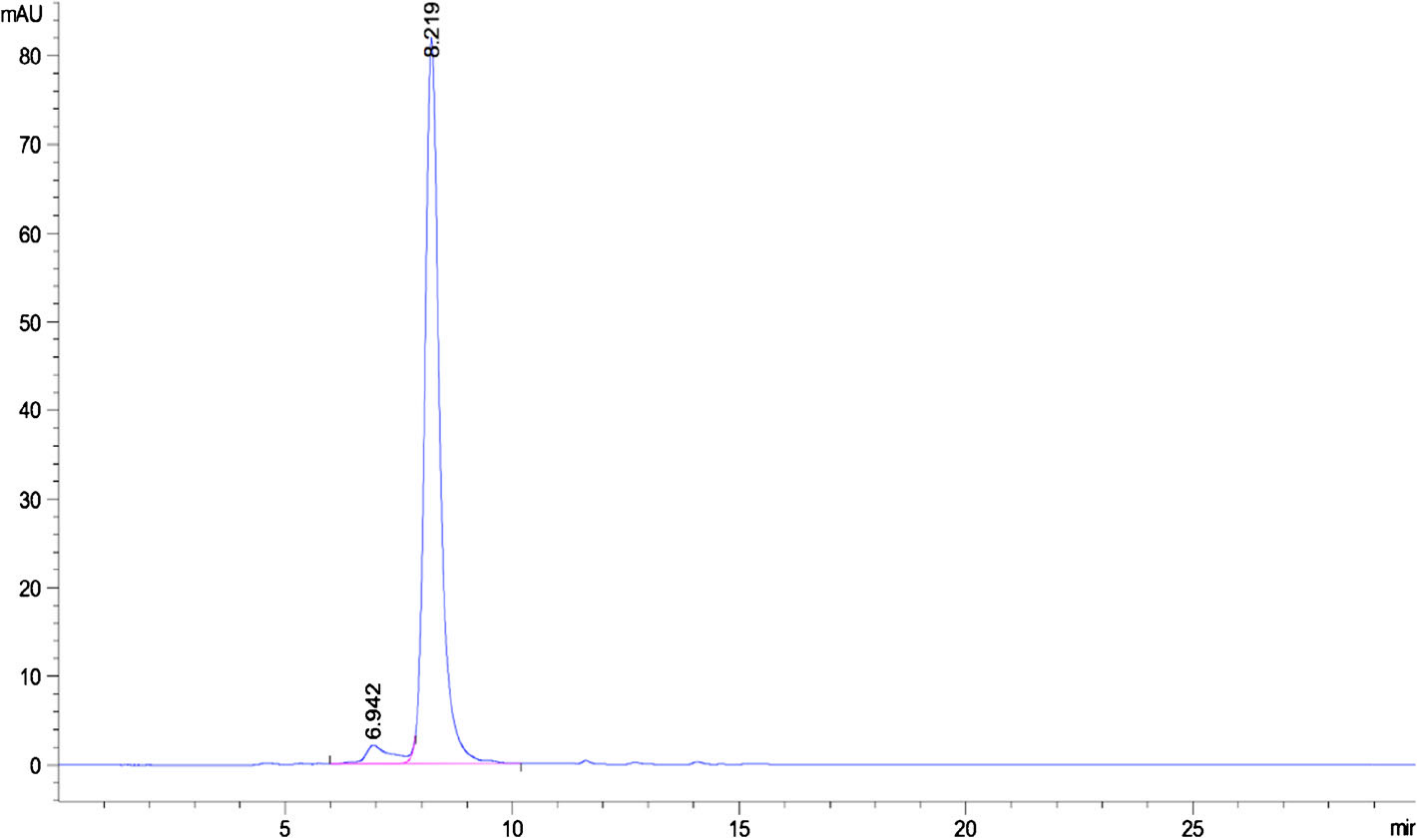
HPLC chromatogram of OVA release from photocrosslinked PEGDA implants.

It is important to confirm that incomplete OVA release cannot be attributed to protein denaturation, thus an ELISA technique was used to determine OVA activity as an alternative to SEC HPLC for protein detection and quantification. SEC HPLC chromatogram shown in Fig. 5 suggests active, non-degraded OVA was released throughout the study, as there was the absence of additional or degradation peaks on the chromatogram. However, the ELISA technique can provide highly specific, sensitive, reliable and rapid results on protein stability and activity and confirm which portion of the protein released is structurally sound (39). From the ELISA results shown in Table it can be clearly concluded that, over the 56-day period, high levels (>90% in all cases) of active protein were continually released. This is a notable finding as current PLGA based implants commonly result in degradation of the encapsulated protein molecules during the release lifetime. A major advantage of this PEGDA based release system is its ability to prevent inactivation of protein over the current release period. This benefit can be attributived to the hydrophilic nature of PEGDA, unlike PLGA, which can commonly cause not only a drop in pH upon degradation (40), resulting in protein denaturation, but also hydrophobic interactions between the polymer and protein molecule hindering drug release (41).

**Fig. 6:**
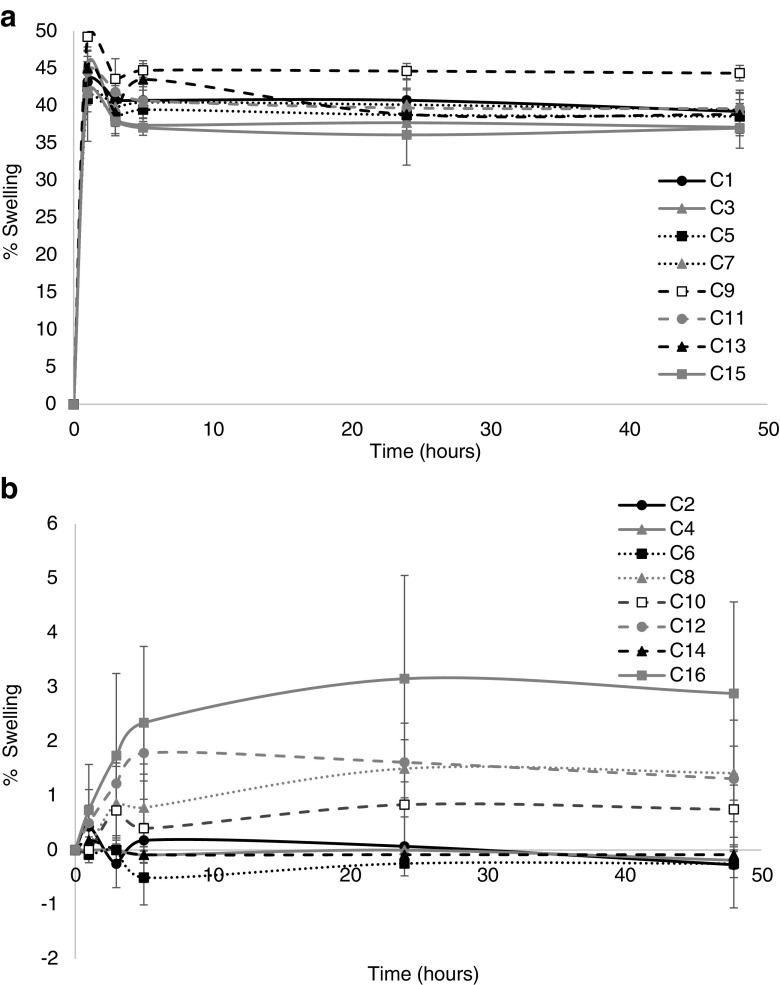
Percentage swelling of (a) PEGDA 700 and (**b**) PEGDA 250 implants. (Mean ± S.D., n = 3).

**Fig. 7:**
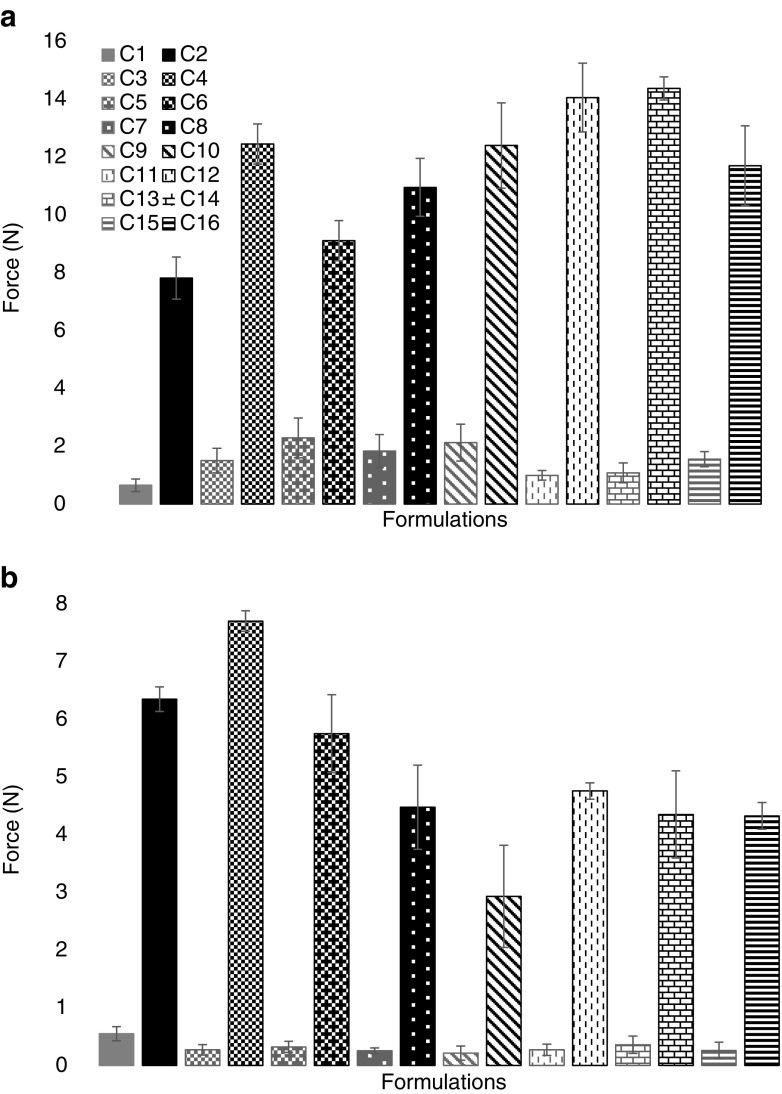
Maximum force (N) required to compress (**b**) dry and (**b**) wet implants of PEGDA MW 700 and 250 5 mm. (Mean ± S.D., n = 3).

**Fig. 8:**
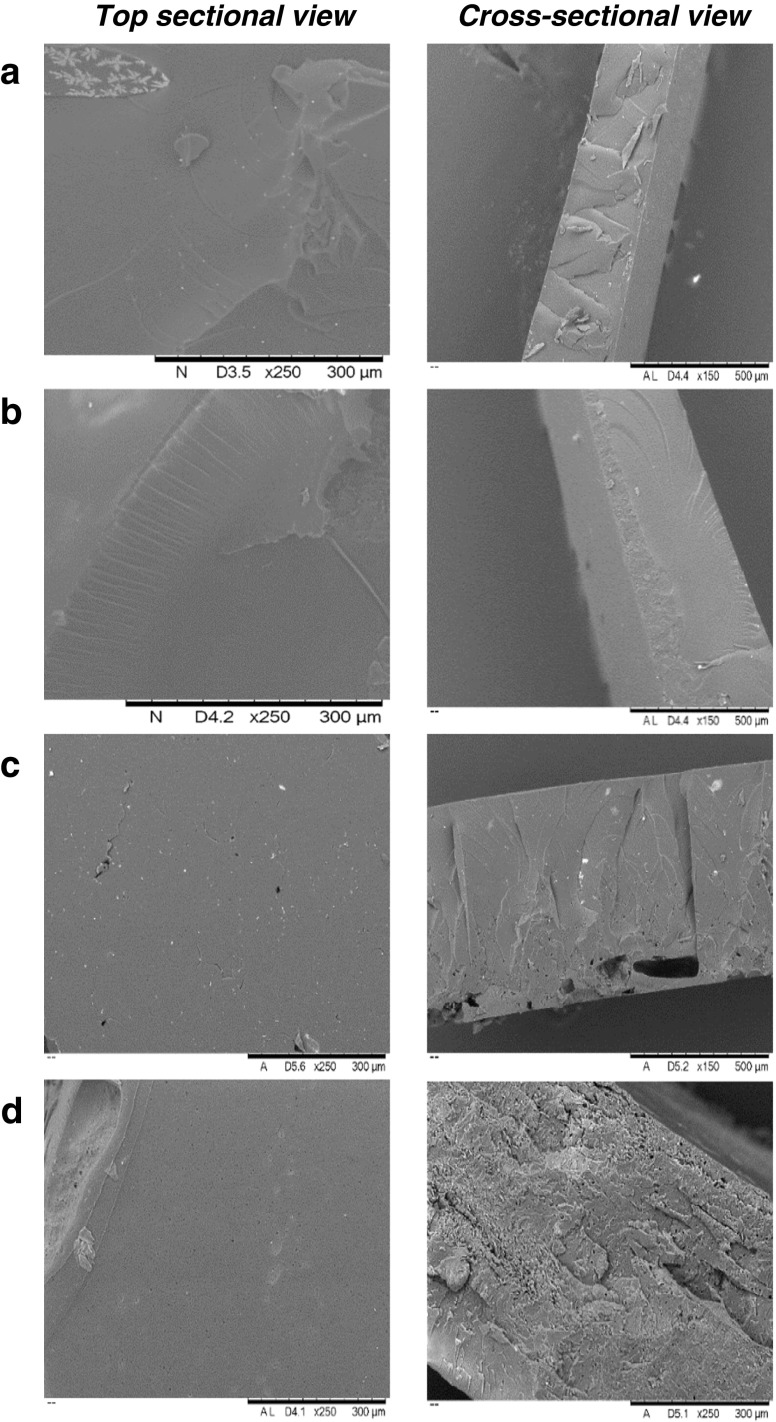
SEM images showing non-drug loaded implants after 24 h in PBS (**a**) C1, (**b**) C2, (**c**) C7, (**d**) C8.

**Fig. 9:**
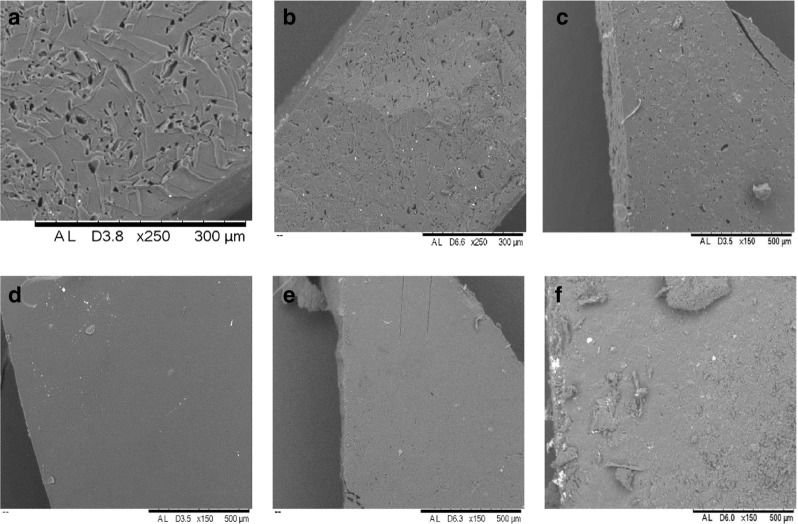
SEM images showing mannitol loaded PEGDA 700 implants after 24 h in PBS (**a**) C3, (**b**) T3, (**b**) O3 and mannitol loaded PEGDA 250 implants after 24 h in PBS, (**d**) C4, (**e**) T4, and (**f**) O4.

**Fig. 10:**
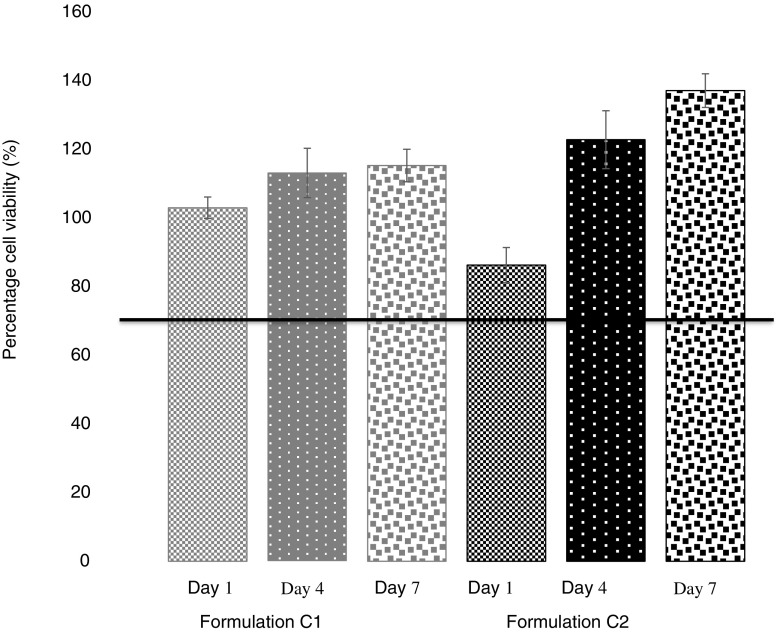
Percentage viability of ARPE-19 cells after 24 h exposure to release media from photocrosslinked implants of Formulation C1 and C2 (Indirect contact assay). (Mean ± S.D., n = 3).

**Fig. 11:**
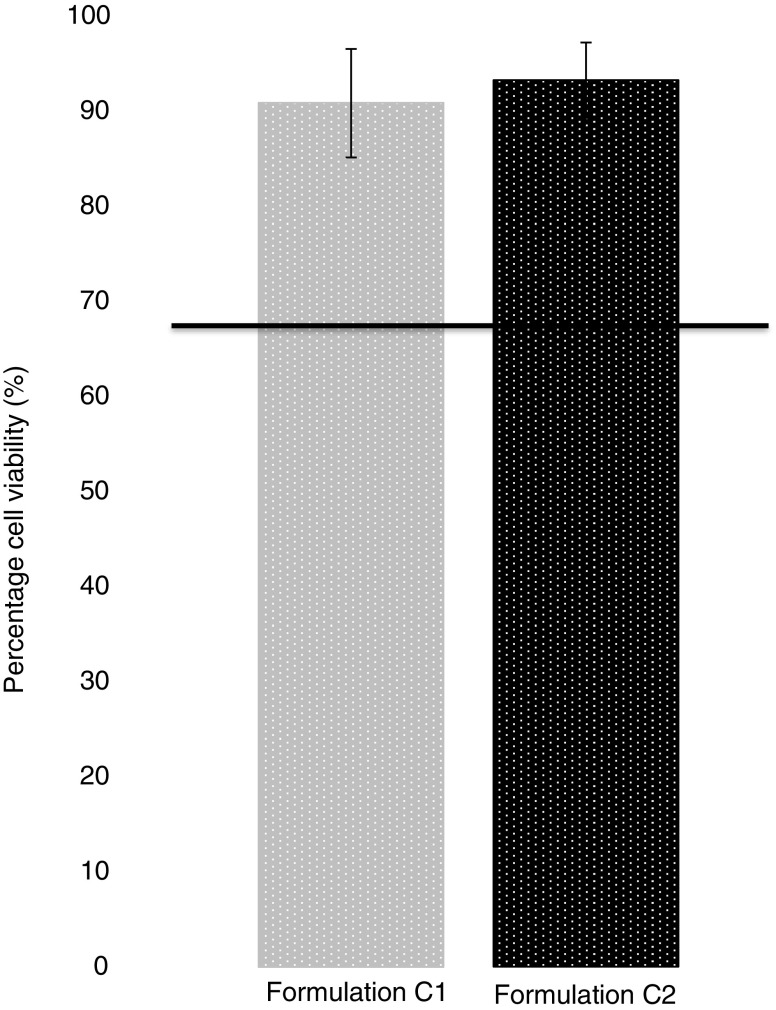
Percentage viability of ARPE-19 cells after 24 h exposure to photocrosslinked implants of Formulation C1 and C2 (Direct contact assay). (Mean ± S.D., n = 3).

**Fig. 12:**
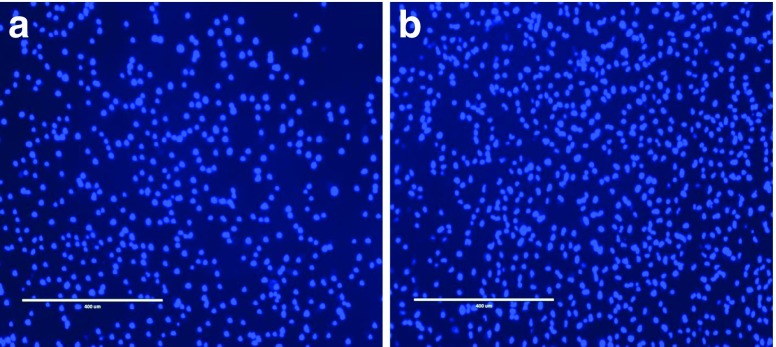
Showing DAPI staining of ARPE-19 cells after exposure to photocrosslinked implants of Formulation C1 and C2 by (**a**) indirect contact assay (**b**) direct contact assay.

Swelling results indicate that higher MW PEGDA implants result in increased system swelling (15). This likely occurs due to high crosslink density within the system when lower MW PEGDA is used, resulting in less space within the network for water ingression and thus swelling potential (42). The addition of pore forming agent on swelling percentage is minimal with both low and higher MW PEGDA systems. As the results stated, no pore-forming agent loaded in PEGDA 700 or PEGDA 250 implants demonstrated a significant difference in swelling when compared to the control formulation (with the exception of gelatin (C9) loaded PEGDA 700 implants). This indicates that polymer MW is the key factor influencing swelling. It is likely that the PEGDA 700 system has reached its maximum swelling capacity and once this is reached the introduction of pore formers does not induce an increase in swelling as the system is saturated. In contrast the PEGDA 250 system is so tightly crosslinked that water cannot access the system and consequently pore forming loading has minimal effect on swelling (36).

From the implant swelling data, network parameters were utilised to assess crosslink density (Ve), polymer-water interaction parameter (χ), average molecular weight between crosslinks (Mc), and mesh size (ε). Network parameters were employed to characterise the complex system structure and evaluate the degree of crosslinking and interaction occurring within the implant formulations (32). As demonstrated by the network parameters, a decrease in polymer chain length results in an increase in crosslink density (Ve) within the system. Indicating that PEGDA 250 implants are more densely crosslinked than their PEGDA 700 counterparts and accounting for the significant differences observed with TA release from the varying MW systems.

Higher χ values for PEGDA 250 implants indicating more polymer-polymer interaction within these systems (32) and thus less polymer-water interactions i.e. increasing crosslinking densities decreases the interaction between the polymeric system and water. This is demonstrated by the swelling data, which shows a drastic reduction in swelling from PEGDA 250 systems when compared with those composed of PEGDA 700. As the results highlighted, formulations composed of higher MW PEGDA resulted in higher Mc and mesh size values, which is due to lower crosslink densities, Ve. By decreasing PEGDA MW, the shorter chained monomer molecules are capable of crosslinking tighter, resulting in the increase in crosslinking densities of these hydrogels. Consequently, decreasing PEGDA MW resulted in more rigid network structures, due to an increased number of crosslinks within the network, which also produced less water ingression into the system and thus the decrease in swelling potential of the lower MW PEGDA systems.

As small alterations in photocrosslinked PEGDA formulation composition have the ability to majorly affect the systems network parameters it is important to ensure that implant mechanical strength is preserved throughout these changes. PEGDA has been shown to be extremely responsive to mechanical modification (43), resulting in hydrogels with flexible properties for drug delivery. Nevertheless, it’s imperative that mechanical strength remains intact to allow implant placement and drug release without substantial bending or breaking of the implant system. Mechanical strength data indicates, that in the dry state, the polymer structures exhibit more rigid polymer chains, and no pores were visible within the structure (although they may be present, but at such a small pore size that they cannot be detected). Due to the lack of visible pores within the implant structure until they are exposed to an aqueous environment, it stands to reason that all dry implants will exhibit greater mechanical strength than wetted implants. The addition of pore former within the implant adds to implant structure and strength when dry, making them harder to compress. In contrast when the implants are subjected to an aqueous environment, for 24 h and mechanical strength is retested, all the wetted samples are consistently weaker than the control of no pore former (with the exception of formulation C4, which still shows increased mechanical strength than the control implant). The decrease in mechanical strength observed in the PEGDA 700 implants, was not statistically significant, however, all PEGDA 250 implants loaded with pore forming agents were significantly weaker than the control (C2) formulation (with the exception of sodium bicarbonate (C8)). Indicating the pore forming agents produce pores within the implant structure on addition to PBS, decreasing the mechanical strength of the implants. When comparing the effect of PEGDA MW on implant mechanical strength, all PEGDA 250 implants are consistently significantly stronger than their PEGDA 700 counterparts, both when dry (*p* < .0001), and upon wetting (*p* < .0001). This is likely due to the smaller MW monomers providing tighter crosslinkage within the system, requiring more force to compress and resulting in an increased mechanical strength.

SEM images support all previously discussed data, with Fig. 8 highlighting the smooth external and internal surfaces of control implants, containing no pore forming agent (C1 and C2) compared to the more porous appearance of those implants loaded with sodium carbonate (formulations C7 and C8). All PEGDA 700 implants are noticeably more porous than the lower MW PEGDA implants in the top sectional image. Suggesting a faster rate of release, increased swelling and lower crosslink densities should be expected from the high MW systems.

Additionally, SEM images, as shown in Fig. 9, help support the hypothesis of a potential interaction between the pore forming agents mannitol and maltose and the OVA protein molecule. Figure 9 highlights the effect of drug loading and drug type on pore formers ability to produce pores within the system. It is clear that the drug type has minimal visual effect on pore formation, further indicating that the decrease in OVA release from mannitol loaded implants is not due to the inability of mannitol (and indeed maltose) to form pores, but rather an interaction or crystallisation of these pore forming agents and the protein molecule preventing release (38). This in turn explains why mannitol loaded TA formulations do not experience the same decrease in drug release that is observed within the OVA formulations.

Biocompatibility studies were conducted using ARPE-19 cells to assess any potential toxicity issues with the formulations. Specifically, there was concern that toxicity could arise from unreacted monomer with the photocrosslinked system (44). Cell viability results shown in Fig. 10 and Fig. 11 indicate that both PEGDA 700 and PEGDA 250 implants were non-toxic to cells. With regards to indirect contact testing, cell exposure to release media collected on day 1 from PEGDA 250 implants resulted in the lowest percentage of cell viability, 86.21%. The ISO standard for tests for *in vitro* cytotoxicity state that a cell viability reading of below 70% is considered a cytotoxic effect (45). Thus, results signify lack of cytotoxicity for all formulations tested. Figure 12 highlights the high portion of live cells (stained blue) present post indirect and direct contact exposure and that the ARPE-19 cells maintain the morphology following exposure. The biocompatibility results are promising and indicate that both the photocrosslinked PEGDA polymer (at MW 250 and 700) and the photoinitiator, Irgacure® 2959, at this concentration are non-toxic to retinal epithelial cells. Photoinitiator selection, along with the polymer used, plays a major role in the crosslinked system that is formed. In this study we use the a type I α-hydroxyalkylphenone (Irgacure® molecule), which is a member of a group of photoinitiator compounds suitable for polymerisation reactions (22). Irgacure® 2959 is an extremely effective type I photoinitiator used in UV photocrosslinked systems (23), and is also suitable for use in aqueous systems or environments (23). Additionally, Irgacure® 2959 is activated by low intensity UV light at a wavelength of 365 nm (24), which is less damaging to tissues and helps further explain its low toxicity. When activated Irgacure® photoinitiators form benzoyl and alkyl radicals, both radicals are reactive to initiate photo-polymerisation but the benzoyl radical presents higher reactivity (46). This free radical formation could have resulted in toxicity, but this study has disproved that. In addition, Williams *et al*. tested a range of α-hydroxyalkylphenones photoinitiators, findings showed that the photoinitiator 2-hydroxy-1-[4-(2-hydroxyethoxy)phenyl]-2-methyl-1-propanone (Irgacure® 2959) exhibited minimal toxicity in mammalian cells lines, including corneal epithelial cells lines (24), and was well tolerated at a range of concentrations, between 0.03–0.1% *w*/w (24,47) supporting findings in this study. This suggests Irgacure® 2959 is a suitable photoinitiator choice and could be safely used in preparations intended for ocular use.

## Conclusion

Photocrosslinked PEGDA implants investigated in this study can provide sustained delivery of both small and large molecules. We have demonstrated that the implant system is capable of releasing bioactive protein molecules over at least a 2 month time period. Additionally, this study shows varying PEGDA MW and/or the addition of pore forming agents can affect drug release from the implant based system and alter its mechanical strength and swelling properties. Thus indicating that this delivery system has the capability to control the drug release by varying the implant composition.

Future work will focus on exploiting PEGDA’s tuneable nature to optimise crosslink density and thus further improve drug release. Different approaches will be investigated for each drug molecule, given their varied physical and chemical properties. The primary aim is to adapt the PEGDA implant system to provide at least 3 months release of active protein, along with desirable mechanical and thermal properties.

## Abbreviations

AMD: Age-related macular degeneration
Da: Daltons
DME: Diabetic macular edema
DR: Diabetic retinopathy
HPLC: High performance liquid chromatography
MTS: (3-(4,5-dimethylthiazol-2-yl)-5-(3carboxymethoxyphenyl)-2-(4-sulfophenyl)-2Htetrazolium)
MW: Molecular weight
OVA: ovalbumin
PBS: Phosphate buffered saline
PEGDA: poly(ethylene glycol) diacrylate
SEC: Size exclusion chromatography
SEM: Scanning electron microscope
TA: triamcinolone acetonide
UV: ultraviolet
VEGF: Vascular endothelial growth factor
